# Parallel Analysis of mRNA and microRNA Microarray Profiles to Explore Functional Regulatory Patterns in Polycystic Kidney Disease: Using PKD/Mhm Rat Model

**DOI:** 10.1371/journal.pone.0053780

**Published:** 2013-01-10

**Authors:** Harsh Dweep, Carsten Sticht, Asawari Kharkar, Priyanka Pandey, Norbert Gretz

**Affiliations:** 1 Medical Faculty Mannheim, Medical Research Center, University of Heidelberg, Mannheim, Germany; 2 Netaji Subhas Sanatorium, National Institute of Biomedical Genomics, West-Bengal, India; University of Torino, Italy

## Abstract

Autosomal polycystic kidney disease (ADPKD) is a frequent monogenic renal disease, characterised by fluid-filled cysts that are thought to result from multiple deregulated pathways such as cell proliferation and apoptosis. MicroRNAs (miRNAs) are small non-coding RNAs that regulate the expression of many genes associated with such biological processes and human pathologies. To explore the possible regulatory role of miRNAs in PKD, the PKD/Mhm (cy/+) rat, served as a model to study human ADPKD. A parallel microarray-based approach was conducted to profile the expression changes of mRNAs and miRNAs in PKD/Mhm rats. 1,573 up- and 1,760 down-regulated genes were differentially expressed in PKD/Mhm. These genes are associated with 17 pathways (such as focal adhesion, cell cycle, ECM-receptor interaction, DNA replication and metabolic pathways) and 47 (e.g., cell proliferation, Wnt and Tgfβ signaling) Gene Ontologies. Furthermore, we found the similar expression patterns of deregulated genes between PKD/Mhm (cy/+) rat and human ADPKD, PKD1^L3/L3^, PKD1^−/−^, Hnf1α-deficient, and Glis2^lacZ/lacZ^ models. Additionally, several differentially regulated genes were noted to be target hubs for miRNAs. We also obtained 8 significantly up-regulated miRNAs (rno-miR-199a-5p, −214, −146b, −21, −34a, −132, −31 and −503) in diseased kidneys of PKD/Mhm rats. Additionally, the binding site overrepresentation and pathway enrichment analyses were accomplished on the putative targets of these 8 miRNAs. 7 out of these 8 miRNAs and their possible interactions have not been previously described in ADPKD. We have shown a strong overlap of functional patterns (pathways) between deregulated miRNAs and mRNAs in the PKD/Mhm (cy/+) rat model. Our findings suggest that several miRNAs may be associated in regulating pathways in ADPKD. We further describe novel miRNAs and their possible targets in ADPKD, which will open new avenues to understand the pathogenesis of human ADPKD. Furthermore they could serve as a useful resource for anti-fibrotic therapeutics.

## Introduction

MicroRNAs (miRNAs) comprise a recently discovered class of small, non-coding RNA molecules of 21–25 nucleotides in length that regulate the gene expression by base-pairing with the transcripts of their targets i.e. protein-coding genes, leading to down-regulation or repression of the target genes [1]. However, target gene activation has also been described [2]. miRNAs are involved in diverse regulatory pathways, including control of developmental timing, apoptosis, cell proliferation, cell differentiation, modulation of immune response to macrophages and organ development [1], [3] and are associated with a wide range of human diseases [3], [4], [5].

Cilia, hair like structures [6], are evolutionary conserved organelles that extend from the cell surface into extracellular space [7] to perform diverse biological functions, including whole-cell locomotion, fluid movement; photo-, chemo-, and mechanosensation and sexual reproduction. Recent findings show an important role of primary cilia in several signal transduction pathways, including Wnt [8], Hedgehog [9], platelet-derived growth factor receptor-alpha (PDGFRα) [10] signaling cascades and cell cycle regulation [11]. The biological importance of primary cilia in human diseases was ignored for a long time; however the dysfunction of cilia and the basal body has recently been recognised in numerous human pathologies (also termed as “ciliopathies” or “cilia-related disorders”) including polycystic kidney diseases (PKD), nephronophthisis (NPHP), Bardet-Biedl syndrome (BBS) and Oral-facial-digital syndrome (OFD). According to the ciliary hypothesis most of the proteins of cystic kidney diseases in humans, rats, mice, or zebrafish are expressed in primary cilia or centrosomes of the renal epithelial cells [7].

Autosomal dominant polycystic kidney disease (ADPKD) is a frequent monogenic renal disease which occurs worldwide with a prevalence of about 1∶1,000 [12]. This is characterised by development of fluid filled renal, hepatic and pancreatic cysts. Nonetheless, the epigenetic factors which regulate cystogenesis are still unknown. Although, accumulating evidence suggests several potential mechanisms such as deregulated cellular proliferation, abnormal programmed cell death (apoptosis), secretion of fluids into the tubular lumen, cyclic adenosine monophosphate (cAMP), irregular extracellular matrix interaction (ECM) and defective planar cell polarity (PCP) could promote cyst formation in ADPKD [13], [14], [15]. For example, a primary defect in apoptotic regulation (e.g. bcl-2 null mice) can result in a cystic phenotype [16]. Moreover, the kidney-specific overexpression of *Myc* leads to a cystic phenotype and an increase in both proliferation and apoptosis [17], [18]. In another study, the cAMP has been shown to play an important role in stimulating cell proliferation and fluid secretion associated with cyst formation in ADPKD [13]. Interestingly, the knocking out of aquaporins (such as *AQP11*) results in cysts formation and finally leads to renal failure, which is similar to PKD [15]. Aberrant increases in basement membrane components (e.g., *Laminin*, *Fibronectin* and *Collagen IV*) have also been described to cause PKD. Due to these changes, the basement membrane could contribute to cyst initiation and expansion [19], [20]. Of note, the key roles of metabolic pathways and their activators have also been reported in the progression of chronic kidney diseases (CKD) in human [21], [22].

Mutant studies have identified some players in PKD development and a transcriptional network involving Hepatocyte Nuclear Factor 1β (*HNF1β*) [23], HNF1α [24], HNF4α and *GLIS2* [25] have been described recently.

**Figure 1:**
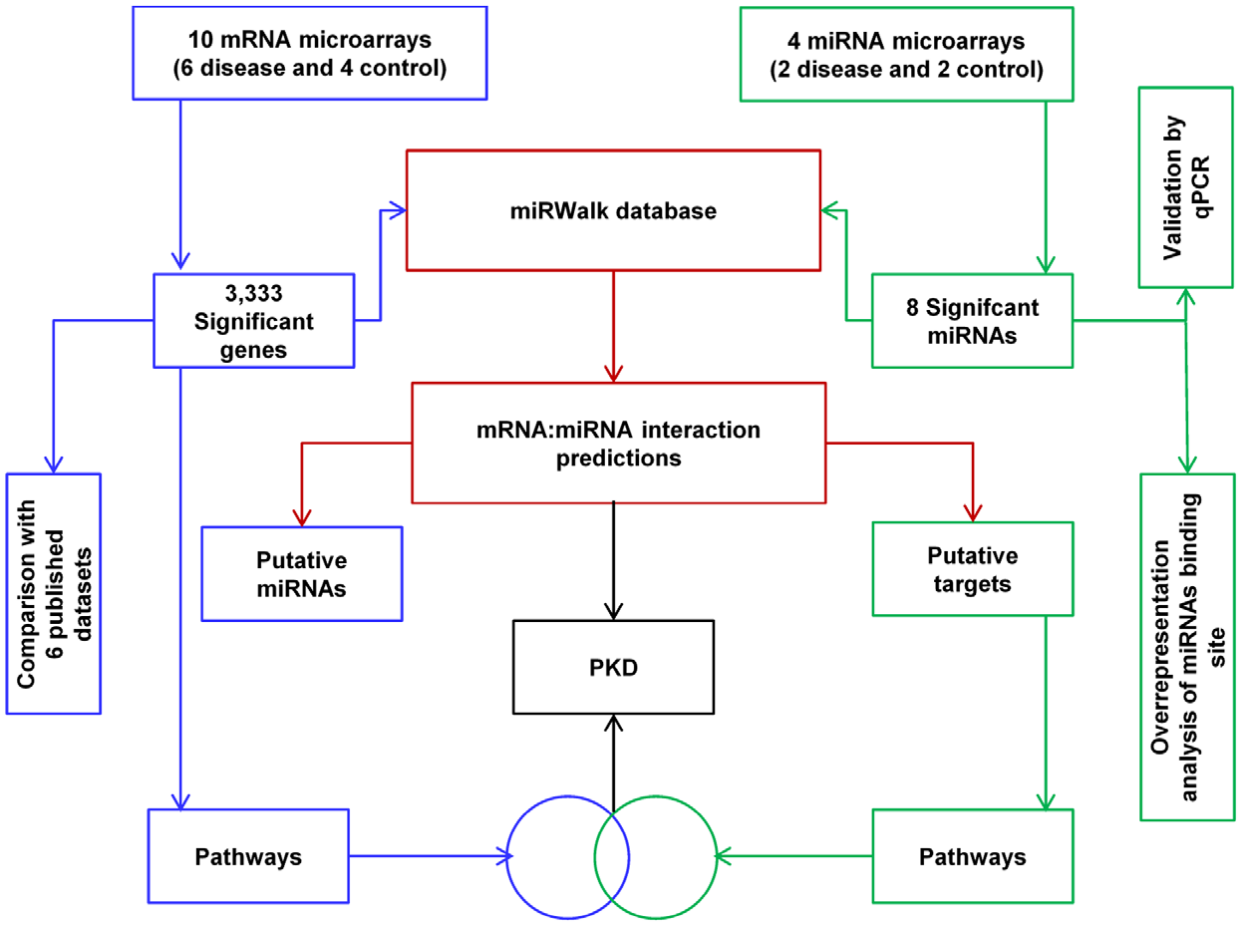
Systematic workflow to explore functional regulatory patterns in PKD.

Rats or mice have been used as common model systems for the study of PKD. The inbred Hannover rat, PKD/Mhm (cy/+), has been efficiently used as a model for human PKD [26], [27]. It is an autosomal dominant model for PKD resulting in cyst formation and slowly progressive chronic renal failure [26], [27]. In the current investigation, (1) we performed microarray profiles using the PKD/Mhm [26], [27] to measure the transcriptional changes (mRNA and miRNAs expressions), (2) comparative transcriptomic studies were conducted to elucidate the similarities with human ADPKD [28], mouse (PKD1^L3/L3^ [29] and PKD1^−/−^ [30]), Hnf1α-deficient [24] and Glis2^lacZ/lacZ^ [25], and (3) in addition we investigated the possible regulatory roles of miRNAs on the expression levels of mRNAs change in PKD/Mhm using miRWalk database [31]. We also conducted miRNA binding site enrichment analysis to identify the overrepresented miRNAs. Furthermore, pathway and Gene Ontology analyses were performed to evaluate enriched functional patterns. We predicted miRNAs that could target significant pathways in ADPKD. **Figure 1** depicts the workflow of this study. Our results suggest that several miRNAs may be involved in regulating the genetic switches in PKD. Additionally, we describe novel possible miRNA:mRNA signatures.

## Methods

### Ethics Statement

An ethical approval was granted by Regierungspraesidium Nordbaden to conduct this experiment on animals.

### Animal Model

After approximately 40 generations of breeding Han:SPRD-cy rats which carry a dominant mutation that causes cystic kidneys and is an accepted model for human PKD, was registered as PKD/Mhm-cy inbred strain of rats: polycystic kidney diseases, Mannheim, Germany hereafter designated as PKD/Mhm. In this study, PKD/Mhm heterozygous (cy/+) and homozygous unaffected (+/+) 36 days old animals, littermates were investigated to profile expression differences in mRNAs and miRNAs. Heterozygous rats develop slowly progressive cystic disease with interstitial fibrosis and thickened basement membranes [32]. About 75% of the cysts are derived from the proximal tubule. The cystic transformation starts with a sharp onset of basement membrane alterations and a loss of epithelial differentiation restricted to small focal areas [26]. There is a substantial matrix overproduction and accumulation of *collagen IV* and *laminin* [26], [33].

The control and diseased animals were sacrificed by cervical dislocation. The left and right kidneys were immediately removed and preserved for histological analyses [34], and the genotypes were confirmed.

### RNA Isolation and Affymetrix mRNA Microarray

Total RNA was extracted using TRIzol method according to manufacturer’s protocol (Invitrogen Life Technologies). cDNA synthesis was performed using the SuperScript Choice System (Invitrogen Life Technologies, Invitrogen Corporation) according to manufacturer’s protocol. Biotin-labeled cRNA was produced using ENZO BioArray HighYield RNA Transcript Labeling Kit. Standard protocol from Affymetrix (Santa Clara, CA) with 3.3 µL of cDNA was used for the in vitro transcription (IVT). Clean-up of the IVT product was done using CHROMA SPIN-100 columns (Clontech, USA). Spectrophotometric analysis was used for quantification of cRNA with acceptable A260/A280 ratio of 1.9 to 2.1. After that the cRNA was fragmented using Affymetrix defined protocol. Labeled and fragmented cRNA was hybridised to 10 Affymetrix Rat 230_2 microarrays for 16 hours at 45°C using Affymetrix defined protocol. Microarrays were washed using an Affymetrix fluidics station 450 and stained initially with streptavidin phycoerythrin (SA-PE). For each sample the signal was further enhanced by incubation with biotinylated goat anti-streptavidin followed by a second incubation with SA-PE and a second round of intensities were measured. Microarrays were scanned with Affymetrix Genechip scanner 3000 controlled by Affymetrix Genechip Command Console (Affymetrix Microarray Suite) software.

### RNA Isolation and Affymetrix miRNA Microarray

Total RNA was isolated using Trizol method as described for mRNA microarrays followed by additional purification using the miRNeasy Mini Kit (Qiagen). RNA was tested by capillary electrophoresis on an Agilent 2100 bioanalyzer (Agilent) and high quality was confirmed. 1 µg of total RNA was Biotinylated using FlashTag Biotin RNA Labeling Kit according to the manufacturer’s protocol (Genisphere LLC). Labeled product was hybridised to 4 Affymetrix miRNA microarrays (Version 1.0) for 16 hours at 48°C with 60 rpm by using a GeneChip Hybridisation oven 640. Microarrays were then washed and stained. Thereafter, microarrays were scanned.

### Statistical Analysis of Microarray Data

Before statistical evaluation, quality control and scatter plot analyses were carried out to minimise bad quality intensity signals, variation during chip hybridisation and separation of groups used for genetic profiling (**Text S1**). Thereafter, the raw fluorescence intensity values were normalised by applying quantile normalisation. Differential mRNA expression was analysed by one-way analysis of variance (ANOVA) [35], using SAS JMP7 Genomics, version 4, from SAS (SAS Institute, Cary, NC, USA). A false discovery rate (FDR) correction was taken for multiple testing with 5% level of significance (α = 0.05). A custom chip definition file (CDF, version 14 with Entrez GeneIDs based gene definitions) was employed to annotate the arrays [36]. Similarly, the miRNA microarrays were analysed using customised “*R script*” (Bioconductor version 2.10 including affy and limma packages) to obtain differentially regulated miRNAs. To verify the results of miRNA microarray, qRT-PCR assays (TaqMan miRNA assays) were performed.

### Reverse Transcription and Quantitative Real-time PCR (qPCR) Analysis of miRNAs

The reverse transcriptase reactions were performed according to “TaqMan MicroRNA Assays” protocol (Applied Biosystems, USA). TaqMan MicroRNA Reverse Transcription kit (Applied Biosystems) was used for cDNA synthesis. The Reverse Transcription (RT) mix was prepared for cDNA synthesis and transferred into a single reaction tube. 2.5 µL diluted RNA (10 ng) was added to each tube. The RT reaction was accomplished in the cycler with different incubations as indicated in the “TaqMan MicroRNA Assays” protocol (Applied Biosystems, USA). Then Real-Time PCR was performed. Thereafter, a master mix was prepared by adding 5 µL universal PCR Master Mix with NO UNGase from Applied Biosystems and 1 µL TaqMan probe. The prepared master mix was aliquotted on the 96-well plate and then 4 µL of the template (cDNA) was added. Cycling conditions were kept as indicated.

The real-time PCR was performed on MxPro – Mx3005P v4.01 Build 369, Schema 80 quantitative PCR software from Stratagene. All the reactions were run in triplicates. The threshold cycle (CT) is defined as the fractional cycle number at which the fluorescence passes the fixed threshold. The expression of miRNA genes between control and PKD/Mhm (cy/+) animals was determined according to manufacturer’s protocol. The mir-193a was chosen as an endogenous control because of its uniform expression in PKD/Mhm [34].

The fold change expression of miRNAs among the two groups was examined by MxPro – Mx3005P software. The significance of differences in relative expression of miRNAs among the two groups was tested by implementing t-test method in ‘R’. The relative expression plots of miRNAs were created by using the same “*R script*”.

### Gene Ontology and Pathway Enrichment Analysis

The DAVID database [37] was utilised to collect the statistically enriched pathways and GO terms information for 1,573 up- and 1,760 down-regulated genes that may modulate cystogenesis.

### Comparison with Other ADPKD Datasets

ADPKD datasets with mutation in PKD1: Pkd1^L3/L3^ [29], human ADPKD [28], Pkd1^−/−^ [30] and Pandey et al [34] were downloaded from 4 published studies. In human ADPKD study, the samples were collected from 5 polycystic kidneys having renal cysts of different sizes: small, medium and large. The size of each cyst was determined by measuring the amount of cystic fluid. The volume cut-off values of cystic fluid for defining the different sizes were set to less than 1 ml, between 10 to 25 ml, and greater than 50 ml for small, medium and large cysts, respectively. All the patients used in this study were shown to have PKD1 by DNA linkage or documentation of pathogenic mutation [28].

The lists of significantly regulated genes of these 4 studies were compared with the differentially expressed genes observed in the cystic kidneys of PKD/Mhm rats. Moreover, the significantly regulated genes in cystic kidneys of PKD/Mhm rats were also compared with two other studies that were conducted on Hnf1α-deficient mice [24] and Glis2^lacZ/lacZ^ model [25].

### miRNA Target Predictions and Enrichment Analysis

An “in-silico” screening of miRNA binding site predictions was carried out within deregulated genes by adopting the miRWalk algorithm [31]. Furthermore, a meta-analysis for miRNA binding site predictions was performed using the comprehensive platform of miRWalk database. The comparative platform of miRWalk database supplies the possible miRNA binding sites resulting from 10 different prediction datasets. Moreover, integrating the results from multiple miRNA-target prediction algorithms can be very helpful in reducing the false positive targets [38], [39]. Thus, the putative targets (genes) information for deregulated miRNAs were downloaded by interrogating the “MicroRNA Target web interface” which is implemented under the “Predicted Target module” of miRWalk database and then the commonly predicted genes were separated. For overrepresentation analysis of miRNA binding site, a customised “R script” was used by implementing Fishers’ exact test with Benjamini and Hochberg (BH) as multiple testing (background correction) with 5% level of significance.

## Results

### Statistical Analysis of mRNA Microarray Profiling in PKD

The expression of 3,333 genes was significantly changed between diseased and healthy kidneys (**Table S1**). We found 1,573 genes as significantly up-regulated, whereas the remaining 1,760 genes were significantly down-regulated in diseased kidneys compared to healthy.

**Figure 2:**
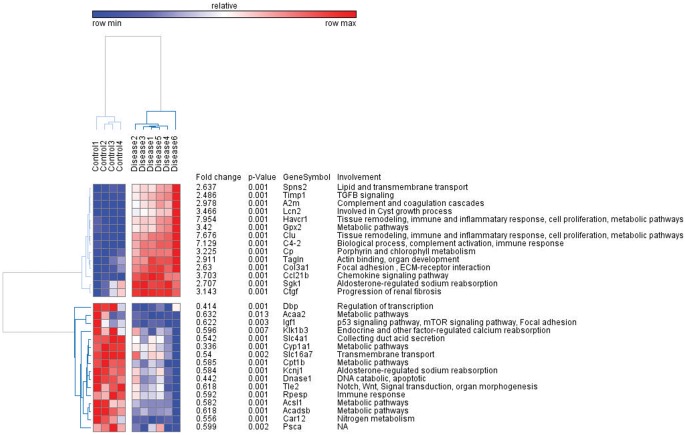
Differential expression of top 30 genes in PKD and control animals. The heatmap was produced by clustering the data matrix of top 30 genes using Pearson correlation. The gene clustering tree is shown on the left and the sample clustering tree is shown on the top. The other information such as fold change expression, p-value, gene symbol and involvement are given on the right. The samples are broadly divided into two groups, healthy (control) and PKD. The color scale shown at the top illustrates the relative expression level of the indicated genes across all samples.

Several markers for the severity of tubular damage such as Havcr1 and *Clu* were found highly expressed in PKD. These genes are involved in tissue remodelling, immune/inflammatory response, cell adhesion, cell proliferation, cell migration and metabolic pathways [37]. **Figure 2** shows the top 30 significantly regulated (15 up- and 15 down-regulated) genes, according to their p-values.

We also observed up-regulation of multiple developmental genes including secreted modulators (*Bmp1*, *Mmp12* and Kcnj8) and integrin receptors subunit (*Itga1*, *5* and *11*) in PKD. Microarray analysis also showed many differentially expressed transcription factors (TFs) such as *Myc*, *Jun*, *Stat3* and *Smad1* were up-regulated, whereas *Sip1, Hnf1α, Hnf4α* and *Glis2* were down-regulated in diseased kidneys.

### Functional Enrichment Analysis of Differentially Regulated Genes in PKD

We obtained 12 and 5 significantly enriched pathways linked with up- and down-regulated genes, respectively (**Table 1**). The most relevant pathways (such as focal adhesion, cell cycle, ECM-receptor interaction, regulation of actin cytoskeleton and MAPK signaling) were found significantly up-regulated in PKD. We also noted 5 significant metabolic pathways on down-regulated genes. Moreover, 45 and 2 GO biological processes were observed to be associated with up- and down-regulated genes, respectively (**Table 2** **)**.

**Table 1:** Differentially regulated pathways on significantly up- and –down-regulated genes.

Pathway	p-value	Fold Enrichment	Benjamini correction
***Pathways associated with significantly up-regulated genes***			
**Focal adhesion**	1.47E−10	2.71	2.41E−08
**Cell cycle**	6.32E−10	3.15	5.18E−08
**ECM-receptor interaction**	3.79E−09	3.68	2.07E−07
**DNA replication**	3.12E−05	4.10	0.001
**Regulation of actin cytoskeleton**	2.12E−04	1.90	0.004
**Fc gamma R-mediated phagocytosis**	2.48E−04	2.51	0.004
**Pathways in cancer**	3.77E−04	1.67	0.005
**Hematopoietic cell lineage**	4.63E−04	2.54	0.005
**Chemokine signaling pathway**	5.93E−04	1.93	0.006
**Leukocyte transendothelial migration**	0.003	2.01	0.032
**Neurotrophin signaling pathway**	0.004	1.92	0.04
**MAPK signaling pathway**	0.004	1.57	0.04
***Pathways associated with significantly down-regulated genes***			
**Valine, leucine and isoleucine degradation**	1.19E−13	5.831178	2.17E−11
**Fatty acid metabolism**	4.22E−07	4.342839	3.84E−05
**Citrate cycle (TCA cycle)**	6.70E−06	4.649392	4.06E−04
**Pantothenate and CoA biosynthesis**	1.00E−04	5.722329	0.006
**Propanoate metabolism**	5.00E−04	3.576456	0.01

**Table 2:** Differentially regulated GO biological processes associated with up- and down-regulated genes.

Gene Ontology Biological Process	p-value	Fold Enrichment	Benjamini correction
***GO biological processes associated with up-regulated genes***			
**Regulation of cell proliferation**	1.76E−09	1.82	3.34E−07
**Positive regulation of developmental process**	3.42E−06	1.96	2.28E−04
**Negative regulation of signal transduction**	4.68E−06	2.23	2.92E−04
**Response to oxygen levels**	1.67E−05	2.14	7.62E−04
**Response to cytokine stimulus**	1.82E−05	2.62	8.03E−04
**Negative regulation of cell communication**	2.20E−05	2.04	9.19E−04
**Regulation of cell cycle**	5.13E−05	1.95	0.001
**Aging**	5.55E−05	2.27	0.001
**Tissue remodeling**	6.42E−05	3.12	0.001
**Actin filament bundle formation**	8.51E−05	5.57	0.002
**Response to mechanical stimulus**	2.01E−04	2.74	0.004
**Intracellular signaling cascade**	2.17E−04	1.39	0.005
**Response to hypoxia**	3.33E−04	1.97	0.007
**Cytokine-mediated signaling**	7.91E−04	2.75	0.014
**Transforming growth factor beta receptor signaling**	0.001	3.06	0.021
**Negative regulation of cell adhesion**	0.001	3.56	0.022
**Myeloid leukocyte activation**	0.001	2.88	0.022
**Transmembrane receptor protein serine/threonine kinase**	0.001	2.40	0.023
**Wnt receptor signaling**	0.001	2.38	0.026
**Microtubule-based process**	0.002	1.80	0.031
**Microtubule cytoskeleton organization**	0.002	2.19	0.033
***GO biological processes associated with down-regulated genes***			
**Oxidation reduction**	4.26E−07	1.70	1.74E−04
**Vesicle-mediated transport**	1.33E−05	1.72	0.002

### Comparison with Other ADPKD Data Sets

We attempted to derive correlations among the gene expression changes in PKD and a set of genes that modulate disease severity in our rat model, and human ADPKD. We compared the significantly regulated genes of PKD/Mhm (cy/+) rat models with data obtained from 3 other published studies which include, PKD1^L3/L3^ [29], PKD1^−/−^ [30] and human ADPKD [28]. A total of 599 (346 and 253 up-, and down-regulated, respectively) dysregulated genes were common between the PKD1^L3/L3^ and PKD/Mhm (cy/+) with similar expression patterns (**Table S2**). The comparison between PKD/Mhm (cy/+) dataset and PKD1^−/−^ data showed 37 common genes (with up-regulated expression) (**Table S3**). In a comparison of our data with human ADPKD dataset, a total of 38 dysregulated genes with up-regulated expression patterns were observed (**Table S4**). Moreover, we obtained 315 up- and 127 down-regulated genes (with similar expressions) when our PKD/Mhm dataset was compared with the data obtained from Pandey et al [34] (**Table S5**). Notably, many pathways (such as focal adhesion, cell cycle, ECM-receptor interaction, regulation of acting cytoskeleton, Mapk and metabolic pathways) were also found as commonly deregulated in both datasets.

### miRNA Microarray Data Analysis in PKD and Validation of the Findings

**Figure 3:**
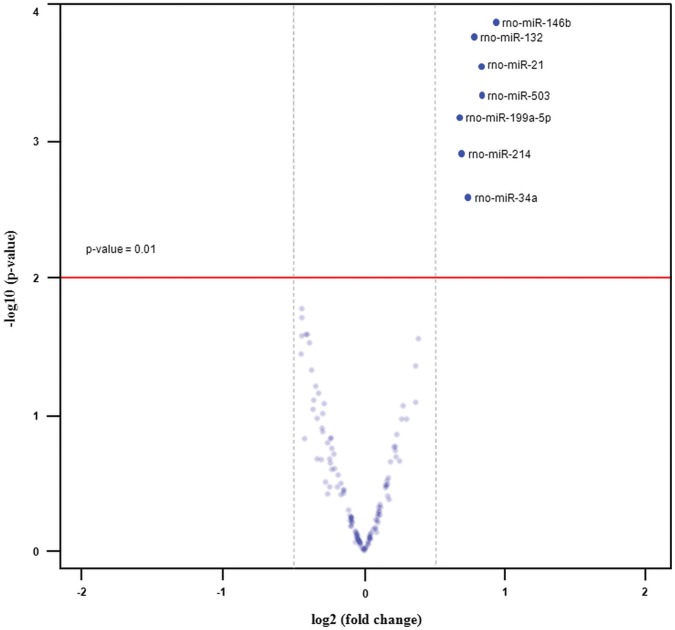
Overview of fold changes of miRNAs versus a measure of statistical significance. The volcano plot shows the -log_10_ (p-value) on the y-axis and the fold change (log_2_) on the x-axis. A cut-off [-log_10_ (p-value) = 1.86] was considered to determine differentially expressed miRNAs between diseased and health (control) animals. Seven miRNAs i.e. rno-miR-146b, -132, -21, -503, -199a-5p, -214 and -34a were found to be significantly up-regulated in diseased animals with a fold change ≥0.5 with significant p-value ≤0.01.

The seven miRNAs i.e. rno-miR-146b, -132, -21, -503, -199a-5p, -214 and -34a were determined as significantly up-regulated in PKD as depicted in **Figure 3**.

**Figure 4:**
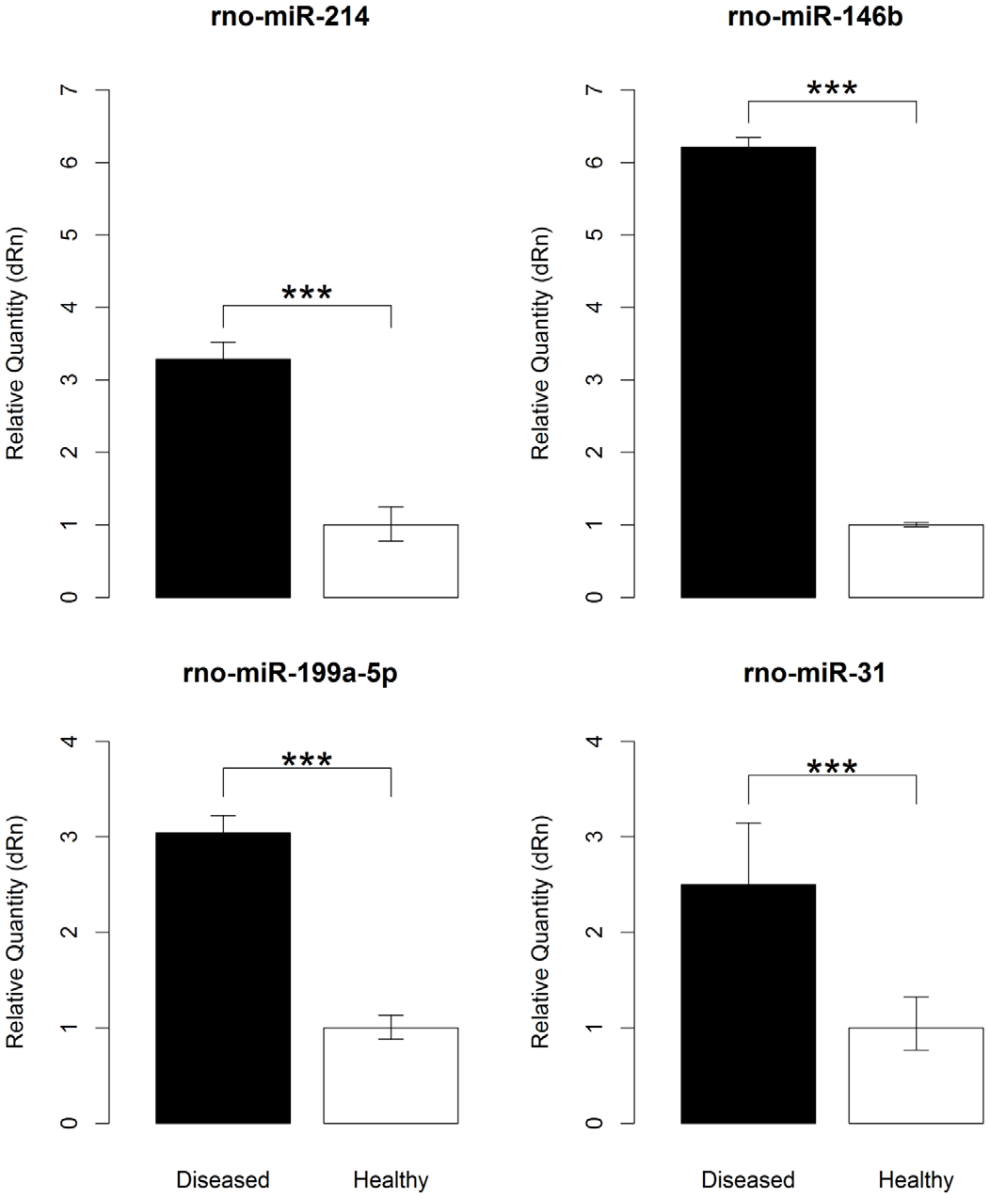
TaqMan assays for miRNAs. The figure shows high abundance of transcripts of rno-miR-146b, -199a-5p, -214 and -31 in PKD/Mhm (cy/+) rat model as observed on microarrays. ‘***’ indicates for p-value <0.0001. The black and white box plots represent cystic and healthy kidneys.

In order to further verify the results of miRNA microarray analysis, we selected three candidates i.e. rno-miR-199a-5p, -214 and -146b miRNAs for quantitative real-time PCR (qPCR) assays. We also chose rno-miR-31 as another candidate for qPCR due to its previous verification in PKD/Mhm (cy/+) rat model [34]. Taqman assays showed increased expression of rno-miR-199a-5p, -214, -146b and -31 in diseased kidneys (**Figure 4**). These results confirmed the outcomes of miRNA microarrays profiling for selected miRNAs (**Figure 3**).

### Putative Targets Identification and Pathway Enrichment Analysis of 8 Up-regulated miRNAs in PKD

Approximately 33,500 miRNA-target interaction pairs were observed between 8 up-regulated miRNAs and all known genes of rat by performing an in-silico screening using miRWalk database. Many genes were identified as the putative target of these 8 up-regulated miRNAs.

Moreover, about 37,306 binding pairs were also obtained on 8 miRNAs by interrogating the comparative platform of miRWalk database [31] (**Table S6**). More than 100 common genes (such as *Stat3*, *Tp53*, *Ccnl1* and *Bmp3*) were predicted as the putative targets of these 8 miRNAs by 6 different algorithms.

To determine the possible roles of these 8 up-regulated miRNAs in different pathways, we selected only those transcripts that were predicted by at least 2 algorithms. Then, the significantly enriched pathways were evaluated using a customised R-script. A total of 107 significantly enriched pathways were found on the putative target genes of these 8 miRNAs (**Table S7**). Of note, 61 pathways (e.g., metabolic, Wnt signaling, adipocytokine signaling, calcium signaling, Tgf-β signaling and cell cycle) showed a strong overlap with the pathways resulting from 3,333 deregulated genes in PKD.

### Possible Interactions Among Deregulated Genes and Up-regulated miRNAs

The miRWalk algorithm was adopted to identify the putative potential binding sites between deregulated genes and miRNAs during PKD. More than 13,880 mRNA-miRNA interactions were observed. The interaction pairs were decreased to 5,472, when a significant threshold cut-off p-value was set to 0.05. **Table 3** shows an overview of binding seeds of 8 miRNAs that were predicted within the different regions of 3,333 deregulated genes. Notably, miR-214 was predicted to anneal with the maximum number of significant genes (1,118 i.e. 583 down- and 535 up-regulated) and its seeds were more specific to down-regulated genes (**Table 3**). The miR-34a was determined as the 2^nd^ highest targeting miRNA which can anneal with 693 genes.

**Table 3:** Distribution of binding sites of 8 miRNAs within 3,333 deregulated genes.

	Down-regulated genes (sites/genes)				Up-regulated genes (sites/genes)			
miRNAs	*5UTR*	*CDS*	*3UTR*	*Enrichment* *(p-value)*	*5UTR*	*CDS*	*3UTR*	*Enrichment* *(p-value)*
**miR-214**	101/71	438/322	268/190	9.77E−15	60/53	412/313	223/169	2.43E−09
**miR-34a**	43/34	249/188	155/124	7.42E−05	41/37	253/189	152/121	2.14E−12
**miR-199a-5p**	33/28	176/152	170/128	0.003	29/24	164/129	139/93	Non-significant
**miR-146b**	35/28	177/128	147/105	1.17E−08	19/16	144/107	116/84	0.003
**miR-503**	46/50	106/119	151/56	0.0002	47/61	63/119	164/55	2E−06
**miR-31**	62/34	146/77	65/109	1.05E−05	67/40	142/48	60/126	8.54E−07
**miR-132**	17/13	71/59	114/94	0.01	19/17	55/42	125/96	0.0007
**miR-21**	9/7	41/34	65/55	Non- significant	9/4	35/27	53/42	Non-significant

To further support these findings, we accomplished a binding sites overrepresentation analysis in which rno-miR-34a (p-value = 2.14e^−12^), -31, -503, -214, -132 and -146b binding sites were observed as significantly enriched within up-regulated genes (**Table 3**), whereas rno-miR-214 (p-value = 9.77e^−15^), -146b, -31, -34a, -503, -199a-5p and -132 were determined as overrepresented within down-regulated genes (**Table 3** **)**. The rno-miR-34a and -214 were noticed as the highly overrepresented miRNAs within up- and down-regulated genes, respectively. These findings support our previous assumption in which we found that rno-miR-214 binding sites are abundantly present within down-regulated genes.

Furthermore, over 8,700 interactions were obtained by querying the comparative platform of miRWalk database (at least 2 algorithms). 33 (17 down-regulated: e.g., *Acsl1*, *Atp2a3*, and *Eif4a2* and 16 up-regulated: such as *Tp53*, *Stat3* and *Il34*) genes were predicted as target of these 8 miRNAs by 6 different algorithms. Moreover, *Fn1* was predicted to be a putative target of miR-146b (5 programs) and miR-503, -214, -31, -34a, -199a-5p, and -132 (2 programs). These results indicate that the expression of 3,333 deregulated genes could be modulated by these 8 up-regulated miRNAs in PKD.

### Impact of 8 miRNAs on Dysregulated Pathways of 3,333 Deregulated Genes

We wanted to explore how many members of overrepresented pathways are the putative targets of these 8 up-regulated miRNAs and how their possible binding sites could promote cysts formation in healthy kidneys. Toward this task, we determined over 700 and 150 putative binding seeds (7 to 12 nt long) of these 8 miRNAs within the different regions of the members engaged with significantly up- and down-regulated pathways. Few examples include; rno-miR-214 can target *Itgb4* (focal adhesion, ECM-receptor and actin cytoskeleton), *Ccl19* (chemokine signaling), *Mcm4* (cell cycle and DNA replication) and *Tbl1x* (Wnt signaling).

**Table 4** depicts a comprehensive atlas of the possible binding sites of these 8 miRNAs within the deregulated members of differentially regulated pathways. Notably, rno-miR-214 was predicted to have maximum number of binding sites within the representative members of down-regulated pathways, whereas, rno-miR-34a was detected as *miRNA hub* having maximum number of interactions within the members of up-regulated pathways.

**Table 4:** Overview of binding site predictions of 8 miRNAs within the representative members of deregulated pathways.

Terms	miR-132	miR-146b	miR-199a-5p	miR-21	miR-214	miR-31	miR-34a	miR-503	N (interaction)
***Up-regulated pathways***									
**MAPK signaling**	5	11	7	3	21	9	16	7	79
**Focal adhesion**	5	8	6	1	18	12	17	7	74
**Actin cytoskeleton**	5	9	3	2	11	6	18	6	60
**Cell cycle**	2	4	6	3	18	7	12	6	58
**Cytokine-cytokine**	2	4	12	2	10	6	12	6	54
**Chemokine signaling**	0	5	5	2	14	6	11	9	52
**CAMs**	11	4	2	2	11	1	7	8	46
**ECM-receptor interaction**	3	3	3	0	14	5	9	3	40
**Wnt signaling**	3	1	2	0	9	6	12	6	39
**VSMC signaling**	1	1	5	1	7	6	7	1	29
**p53 signaling**	2	0	2	1	9	2	8	3	27
**Calcium signaling**	0	3	4	1	4	2	7	2	23
**Apoptosis**	1	2	3	2	5	3	5	2	23
**DNA replication**	1	0	2	3	7	2	5	2	22
**Jak-STAT**	3	2	3	1	5	2	3	2	21
**TGF-beta**	0	0	4	0	4	3	4	1	16
**Phosphatidylinositol**	0	2	6	0	3	2	2	0	15
***Significantly down-regulated pathways***									
**V.L.I. degradation**	9	6	8	4	8	4	9	6	54
**Fatty acid metabolism**	4	2	6	3	7	1	6	3	32
**Propanoate metabolism**	6	2	5	3	3	2	2	2	25
**Citrate cycle (TCA cycle)**	2	4	2	1	5	1	2	1	18
**Pantothenate and CoA**	0	5	1	1	5	1	4	1	18

## Discussion

Parallel mRNA and miRNA microarray profiling in PKD revealed 3,333 significantly deregulated genes and 8 up-regulated miRNAs, respectively. The expression patterns of nephron segment-specific genes (e.g., *Pck1*, *Kcnj1a* and *Scnn1g*) were down-regulated. Of note, we found up-regulation of multiple developmental genes (e.g., *Bmp1*, *Mmp12* and *kcnj8*), transcription factors (TFs) (e.g., *Myc*, *Ap1*, *Stat3* and *Cux1*) and integrins (e.g., *Itga1*, *2b*, *a3*, *5* and *b11*). Many of these members are closely related to ureteric bud formation, outgrowth and branching during metanephric kidney development [28], [29], [40]. On the other hand, up-regulation of genes i.e. *Bmp1*, *Tgfβ1*, *Tgfβ2*, *Tgfβr1* and *Tgfβr2* suggest epithelial-mesenchymal transition (EMT). Taken together, these data suggest re-activation of signaling pathways for renal development and repair in PKD/Mhm renal cysts. Further, the previous studies of the cystic kidneys implicated TGF-β in renal cell hypertrophy and extracellular matrix (ECM) deposition in diabetic nephropathy [41] and PKD [42]. The expression of ECM components such as *Cthrc1*, *Fn1* and *Collagens* was significantly higher in PKD.

*MYC* gene is often found up-regulated in human ADPKD [17] and we also detected the similar expression in our study. *MYC* is known to be involved in cell proliferation, apoptosis, differentiation and neoplasia [18]. Of note, in the kidneys of PKD patients, both apoptosis and proliferation are associated with increased expression of *Myc* [43].

Interestingly, *Pla2g2a*, *Pla2g4a* and *Ptgfrn* genes (involved in cAMP mediated signaling and calcium regulation) were also up-regulated, while negative regulators (such as *Rgs9*, *Rgs12* and *Rgs14*) of GPCR signaling were down-regulated. These transcriptomic changes suggest a modification in GPCR signaling that may result in the regulation of cAMP and calcium signaling in PKD. We also observed up-regulation of *Adcy2*. This gene may elevate the cAMP signaling in cysts which further leads to proliferation [44]. An increased in the cAMP production has previously been shown to stimulate renal cystic epithelial proliferation in PKD [44].

Notably, the additional genes (down-regulated: *Nphp1*, *Mks1* and *Bbs4*; up-regulated: *Pkd2*, *Nphp4*, *Bbs7*, *Ofd1* and *Pdgfra*) associated with ciliary function or renal cystic disorders were also differentially expressed in diseased kidneys. Abnormal expression of *Pkd2* leads to disrupt multiple biological processes (e.g., cell division, proliferation, and deregulated cell-matrix and/or cell-cell interactions). The up-regulation of similar processes such as cell cycle, proliferation, ECM-receptor interaction and focal adhesion were also noticed in PKD.

Moreover, 12 pathways (such as focal adhesion, cell cycle and ECM-receptor interaction) were identified as significantly up-regulated in PKD. Previous studies suggested that an aberrant activation of these pathways plays a role in ADPKD. Therefore, these genes might be considered as potential markers for the activation of complex network processes that may promote cysts in PKD animals. Additionally, we also obtained five significantly down-regulated metabolic pathways (**Table 1**).

miRNAs have emerged as mediators of gene expression. They direct the regulation of cellular processes include; differentiation, proliferation, cell-cell interaction, cell death, metabolism and many diseases. Another important aim of this study was to determine the possible regulatory role of miRNAs in ADPKD. Several potential binding sites were observed between 3,333 deregulated genes and 8 up-regulated miRNAs in PKD using the miRWalk database [31]. A few potential interactions are: rno-miR-214, -31, -199a-5p, -21, -34a and -132 were predicted to target several down-regulated TFs such as *Hnf1α*, *Nfatc4* and *Glis2*. These TFs may play a crucial role (either positive or negative regulators) in the promoter region of deregulated genes in the cystic kidneys. Our findings show an agreement with a previous study that states miRNAs often control the expression of key genes by annealing within the sequence of upstream flanking regions’ regulators (repressors or enhancers) [45].

The rno-miR-199a-5p/214 transcript was previously reported during development [46] and epithelial ovarian cancer cells [47]. The implications of miR-146a (but not miR-146b) have been shown in chronic renal inflammation [48], human renal cell carcinoma [49] and retinoic acid induction in acute promyelocytic leukemia [50]. The miR-21 has been reported to promote mostly fibrosis [51], to correlate lower kidney cancer survival [52] and to modulate cell apoptosis in renal cell carcinoma [53]. The rno-miR-31 is the only miRNAs that has been previously reported in PKD [34]. Of note, 7 of these 8 miRNAs have not been previously reported in PKD.

miRNAs direct the temporal changes in the expression of the representative members of key signaling pathways, as these small changes may alter the responsiveness of cells to signaling molecules (such as TGF-β, Wnt, Notch, and EGF) [54]. Once an inhibitor (e.g., *Hnf1α*, *Hnf4α*, *Glis2* and *Sip1*) of signaling networks is down-regulated by one or more miRNAs, these miRNAs then act as a positive regulator which further amplifies the expression of many genes engaged with human pathologies. Such possible regulatory roles were also observed in PKD. For example, the putative seeds of miR-199a-5p (9nt long seed) and miR-34a (7nt long seed) within 3′-UTR region of *p27* (*Cdkn1b*) suggest the possibility that this gene may be down-regulated by these 2 miRNAs in PKD. The *p27* is a CDKs (cyclin-dependent kinases) inhibitor of the cell cycle at G1 phase (*Cdk2*/*Ccne2* complex). Furthermore, *Apc* (down-regulated) was predicted as a putative target of miR-214 with a seed of 10nt long. *Apc* (an E3 ubiquitin ligase) gene stimulates the cell cycle progression by degrading checkpoint proteins such as *Ccnb1* (up-regulated). Moreover, three miRNAs (miR-199a-5p, -214 and -132) were predicted to anneal within 3′-UTR of *Rb1* (down-regulated) which is also a cell cycle inhibitor. Several other down-regulated genes such as *Hes6* (inhibits cell proliferation), *Rbl2* (tumour suppressor and inhibitor of *E2F* target genes) and *Id1* (a bHLH transcription factor that regulates cell proliferation by supressing *p21* or *Cdkn1a*, a CDKs inhibitor) were also observed as the putative targets of rno-miR-503, -214 and -146b and -503. Of note, *Ift88* and *Ift122* were determined as the possible targets of rno-miR-199a-5p and -214. These genes were noticed as significantly down-regulated in PKD. It has been previously shown that a small interfering in *Ift88* accelerates cell cycle progression with an increase in proliferation (reviewed in [55]). Our putative findings suggest that these 8 miRNAs could mediate the down-regulation of the crucial checkpoint genes (inhibitors) of the cell cycle, and proliferation and may result in the aberrant activation of these processes which may lead to PKD. Moreover, the importance of apoptosis has been highlighted in which the inactivation of apoptosis inhibitors such as *Bcl2* causes CKD [16]. 3 miRNAs (miR-34a, -21 and -503) were predicted to target *Bcl2* gene (down-regulated). These 3 miRNAs may decrease the expression of *Bcl2* and could cause PKD via abnormal activation of apoptosis. Interestingly, other down-regulated genes that encode for cilia and PCP signaling proteins such as *Tsc2*, *Nek9*, *Bbs4*, *Mks1*, *Dvl1*, *Pkd2*, *Sod2* and *Wnt10a* were also determined as the putative targets of these 8 up-regulated miRNAs. Altogether, it can be concluded that these mRNA:miRNA interactions may result in ADPKD via the dysregulation of cell cycle, differentiation, orientation, proliferation and apoptosis. The abnormalities in these pathways have previously been reported in human ADPKD [17], [56], [57].

Interestingly, several potential interactions between 8 up-regulated miRNAs and 6 down-regulated aquaporins (AQPs) genes were observed for examples: miR-199a-5p, -214 and -503 can anneal to *Aqp1* and *Aqp12a* with octamer seeds; and *Aqp4* has multiple 9nt long binding sites for miR-132. These binding sites may explain why the kidneys of PKD/Mhm rats have enlarged cysts. Since, AQPs are the most important water channel for transepithelial water permeability, and exit pathway for water that enters the cell [58] and most importantly their knocking out resulted in renal failure, which is similar to PKD [15], [59], [60]. These miRNA-mediated down-regulations could abolish these exit doors. Notably, these interactions have not been previously described in ADPKD.

Also, miR-214 and -503 were predicted to hybridise with Peroxisome proliferator-activated receptor α (*Pparα*) (down-regulated) with octamer seeds. *Pparα* is a major TF that regulates a number of lipid oxidation and metabolism pathways. Moreover, many genes (such as *Acad10*, *Acox3*, *Cpt2*, *Dhrs4* and *Sult5a1*) regulated by *Pparα* were also found down-regulated on mRNA-chips and predicted targets of 8 miRNAs. These genes are closely engaged with lipid and other metabolic pathways. It has been previously described that *Pparα* plays a central role in controlling epithelial signals to the interstitium that promote fibrosis via inhibiting the appearance of myofibroblasts as directed by α smooth muscle actins (e.g., *Actn1*, *Actb* and *Actg2*) and inhibiting the development of interstitial fibrosis [51]. The miRNA-mediated down-regulation of *Pparα* and alteration in lipid metabolism pathways could lead to kidney injury and fibrosis as observed in PKD. Furthermore, recent studies identify metabolic pathways and genes regulating them as significantly and independently linked with the progression of CKD [21], [22].

Most, but not all types of CKD are complicated by an excessive accumulation and deposition of ECM that progressively leads to destruction of functional nephrons (tubulointerstitial fibrosis). The miRNA-mediated inhibition of E-Box repressors (e.g., *Zeb1*) of upstream flanking region of fibrotic genes (such as *Fn1* and *Collagens*) under chronic Tgfβ signaling has been demonstrated to drive renal fibrosis [61]. Similarly, 5 miRNAs (e.g., miR-214, -31 and -34a) were predicted to target down-regulated genes such as *Glis2* and *Hnf1α*. A small interference in the expression patterns of these inhibitors may result in ECM deposition and could lead to fibrosis as described in previous studies (associated with PKD). For example, a renal-specific inactivation of *Hnf1α* in mice has been implicated in polycystic kidney disease and shown to mediate the down-regulation of several cystic disease genes localised to primary cilia, including *Ift88* and *Ift122* [23]. On the other hand, *Glis2* has been described as a transcriptional repressor for several genes during the expression profiling of kidneys between wild type and Glis2^lacZ/lacZ^ mice [25]. Moreover, the development of fibrosis in Glis2^lacZ/lacZ^ mice accompanied by increased synthesis of various ECM components, such as various types of *collagen*, *Ltbp2*/*4*, *Fn1* and *vimentin* (*Vim*) has also been described [25]. Furthermore, a comparison of human ADPKD and PKD/Mhm datasets was conducted in which 38 genes were identified with similar expression patterns (**Table S4**). These genes are the representatives of ECM-receptor interaction, focal adhesion, cell cycle, proliferation and metabolic pathways. Also, 174 genes were found with common expression patterns in a comparison with Glis2^lacZ/lacZ^ dataset (**Table S8**). Moreover, 117 genes were noticed with similar expression patterns (87 up- and 30 down-regulated), when the dataset of our ADPKD model was compared with the dataset of Hnf1α-deficient mice [24] (**Table S8**). Taken together, this data suggest a direct transcriptional hierarchy in which *Hnf1α* and *Glis2* regulate other cystic genes. It can be assumed that these 8 up-regulated miRNAs may promote renal fibrosis by targeting the upstream E-Box repressors of up-regulated genes in PKD. Therefore, these discoveries postulate a possibility by which the down-regulation of these 8 miRNAs may restore the normal expression of the crucial members of the altered pathways (such as focal adhesion, cell cycle, regulation of actin cytoskeleton, ECM-receptor interaction and metabolic pathways). Then, these pathways could be stabilised to their normal function by using antagomirs [62].

### Conclusions

In this study we have performed mRNA and miRNA microarray profiles to explore further epigenetic factors which regulate cystogenesis in PKD. Taken together, our microarray data identified multiple genes that are significantly de-regulated during disease condition. Furthermore, our rat model describes up-regulation of several biological processes and down-regulation of metabolic pathways which are closely associated with cyst formation and expansion. We also observed up-regulation of secreted modulators and integrin receptors subunit. In addition, we also detected many differentially regulated TFs in PKD. Interestingly, the down-regulation of genes associated with ciliary function or renal cystic kidney diseases were also differentially regulated. Moreover, we also contribute 8 miRNAs to the complex regulatory layers of ADPKD. We predicted that several deregulated genes/pathways are the targets of these 8 miRNAs. Therefore, we suggest that these 8 miRNAs may play a central role in PKD via the down-regulation of key checkpoint genes (inhibitors) of the crucial pathways. These miRNAs should be explored as novel therapeutic targets by performing knockdown/knockout studies using antagomirs that may stabilise the deregulated pathways to their normal function. Moreover, these miRNAs could serve as useful candidates for anti-fibrotic therapies.

## Supporting Information

Table S1 **Significantly regulated genes found in the diseased kidneys of PKD/Mhm rat model.** This table shows significantly regulated genes on the Affymetrix mRNA microarrays obtained from SAS analysis. A threshold cut-off value was set to 1fold change which indicates that the expression of a given gene is uniform in both diseased and control kidneys. The fold change values greater than 1 indicate up-regulated genes, whereas, the remaining fold change values describe down-regulated genes in diseased kidneys.
(XLS)

Table S2 **Comparison among PKD/Mhm and PKD1^L3/L3^ datasets.** This table shows the common genes obtained by comparing PKD/Mhm rat and PKD1^L3/L3^ models. Similar cut-offs were chosen (as described in Table S1) to display up- and down-regulated genes among these two datasets.
(XLSX)

Table S3 **Comparison between PKD/Mhm rat and PKD1^−/−^ datasets.** This table shows the common genes obtained by comparing PKD/Mhm rat and PKD1^−/−^ models. Similar cut-offs were chosen (as described in Table S1) to display up- and down-regulated genes among these two datasets.
(XLSX)

Table S4 **Comparison between PKD/Mhm rat and human ADPKD datasets.** This table shows the common genes obtained by comparing PKD/Mhm rat and human datasets. Similar cut-offs were chosen (as described in Table S1) to display up- and down-regulated genes among these two datasets.
(XLSX)

Table S5 **Common genes obtained between PKD/Mhm rat data and Pandey et al dataset.** This table shows the common genes obtained by comparing PKD/Mhm rat data with the data obtained from Pandey et al [34]. Similar cut-offs were chosen (as described in Table S1) to display up- and down-regulated genes among these two datasets.
(XLSX)

Table S6 **Comparison between PKD/Mhm rat and 2 published studies.** This table shows the common genes obtained by comparing PKD/Mhm rat and Hnf1α-deficient [24] and Glis2^lacZ/lacZ^ [25] datasets. Similar cut-offs were chosen (as described in Table S1) to display up- and down-regulated genes among these two datasets.
(XLSX)

Table S7 **Putative miRNA binding sites of 8 miRNAs within 3,333 deregulated genes using miRWalk database.** This table shows the putative miRNA binding site predictions obtained from the comparative platform of miRWalk database which integrates the results from 9 different predictions datasets for comprehensive view.
(XLSX)

Table S8 **Significant pathways obtained on 8 up-regulated miRNAs.** This table shows significantly regulated pathways obtained on the target genes of 8 up-regulated miRNAs.
(XLSX)

Text S1 **Information on mRNA and miRNA microarrays quality control analysis.**
(PDF)
