# Supplementary material for: Parallel Analysis of mRNA and microRNA Microarray Profiles to Explore Functional Regulatory Patterns in Polycystic Kidney Disease: Using PKD/Mhm Rat Model
Source: PLoS One. 2013 Jan 10;8(1):e53780. doi: 10.1371/journal.pone.0053780 (PMC3542345; doi:10.1371/journal.pone.0053780)
Supplement: Text S1 — Information on mRNA and miRNA microarrays quality control analysis. (PDF) [file pone.0053780.s009.pdf]

Supplementary Information

mRNA microarray quality control analysis

Scatter plot of intensities between 10 mRNA arrays and their correlation coefficients

Figure 1 provides detailed scatter plots between 10 mRNA microarrays (4 Control and 6 Disease) comparisons along with their correlation coefficients. The maximum correlation i.e. 0.996 and 0.998 are obtained between Control3 vs Control4 and Disease1 vs Disease5, respectively, whereas the lowest correlation is observed between Control1 and Disease6. Thereafter, a hierarchical clustering analysis was applied to view the separation of these 10 chips. The result of clustering analysis is shown in **Figure 2** which indicates that all 10 chips are very well separated in two different groups i.e. Disease and Control. The results obtained from scatter plot and hierarchical clustering illustrate that the chips were free from systematic biases and were ready for further statistical evaluation.

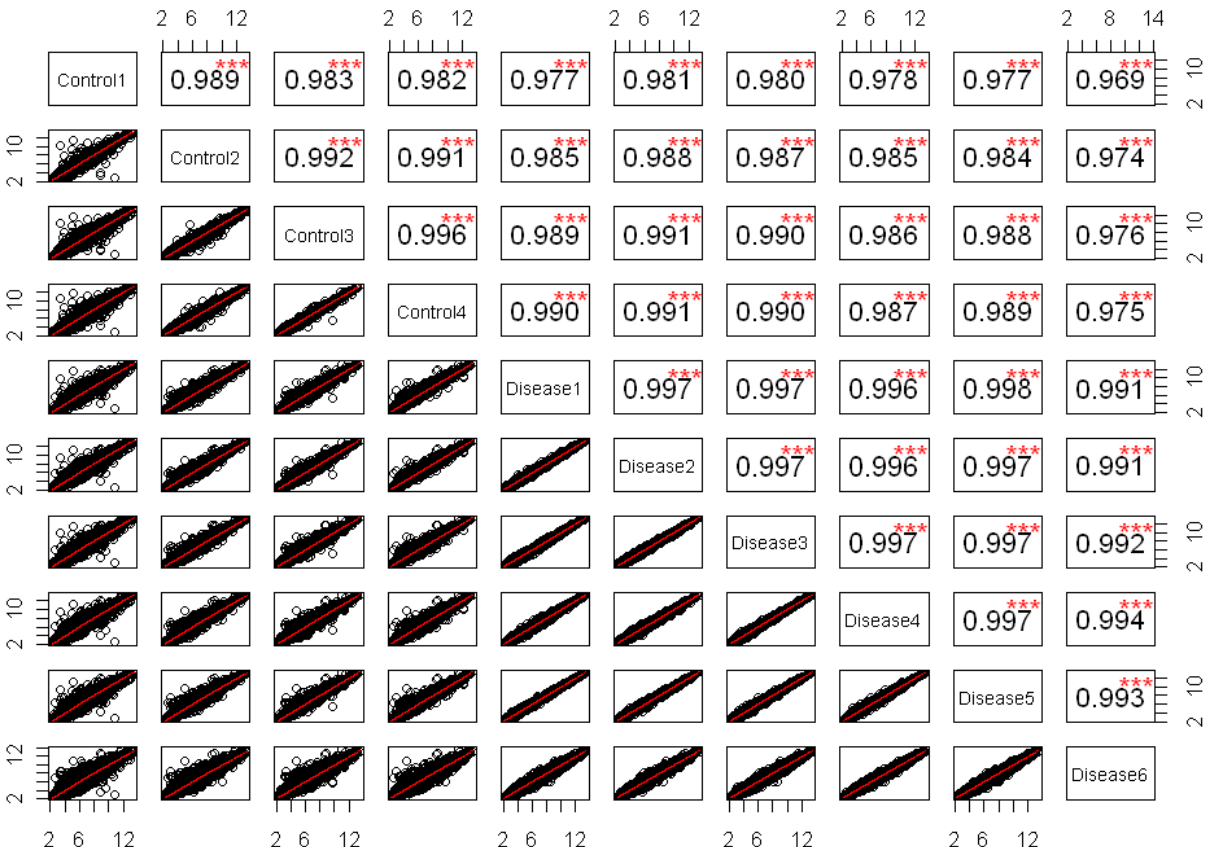

Figure 1: Scatter plots of all pairwise comparisons of probe intensities among 10 mRNA microarrays.

The upper and lower triangles of each square show the probe intensities along with regression estimation bisector lines and correlation coefficient among each pair. The maximum correlations were observed among Disease1 vs Disease5 and Control3 vs Control4 as expected. The highest correlation was found among Control3 and Control4 arrays.

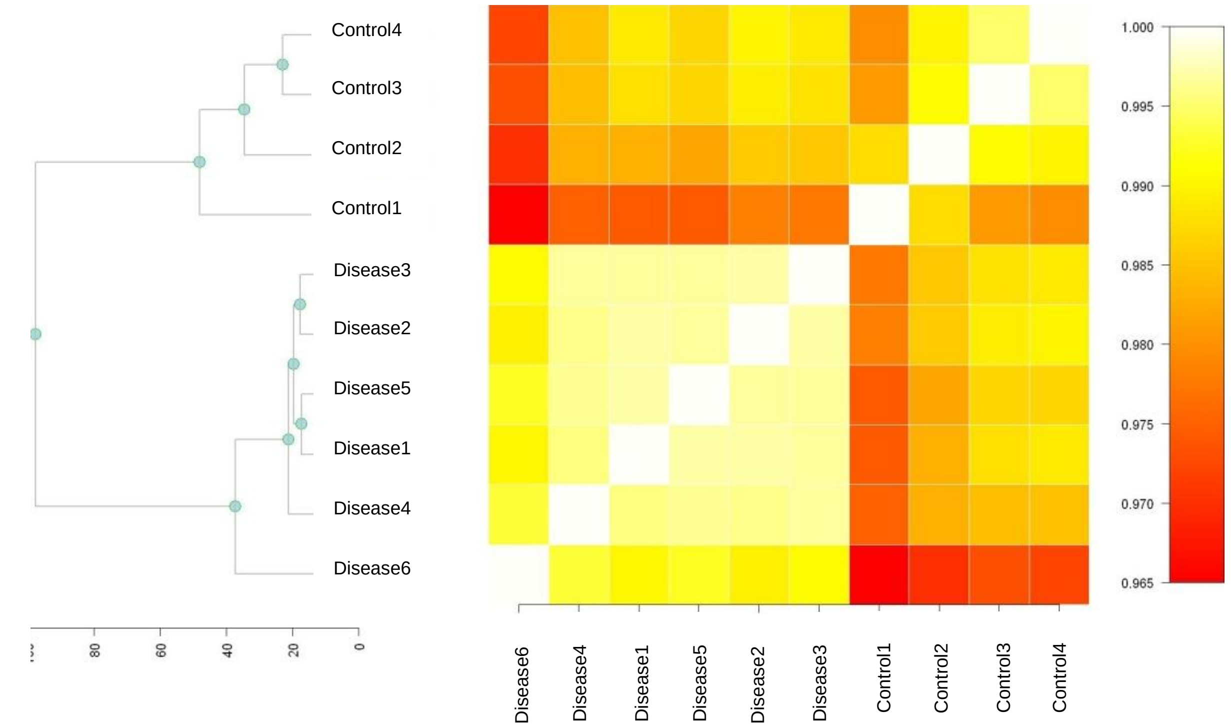

Figure 2: Hierarchical clustering analysis heat map of 10 mRNA microarrays from 10 different animals.

The arrays are well separated into two different groups. The two major branches of the dendrogram represent disease and control animals with a correlation of 0.987 and 0.990 respectively. The high correlations values indicate that these samples were free from systematic biases.

miRNA microarrays quality control analysis

Scatter plot of intensities between four miRNA chips and the correlation coefficients

Figure3 provides detailed scatter plots of 4 miRNA chips comparison. Moreover, the correlation coefficients were evaluated for each of the four chips. For a given chip i.e. Diseased1, Diseased2, Healthy1 or Healthy2, all pairwise correlations between four chips were computed. The maximum correlations observed are 0.987 and 0.990 for Diseased1:Disease2 and Healthy1:Healthy2, whereas the smallest correlations are observed between Disease and Healthy chips. No bad quality of the chips was identified. Thereafter, a hierarchical clustering analysis was applied to view the separation of 4 arrays. The result of clustering analysis is shown in **Figure 4** which shows that all the four chips are well clustered in two group's i.e. disease and healthy. The values obtained from scatter plots correlation and hierarchical clustering heat maps denote that the chips were free from systematic biases and were ready for further analysis.

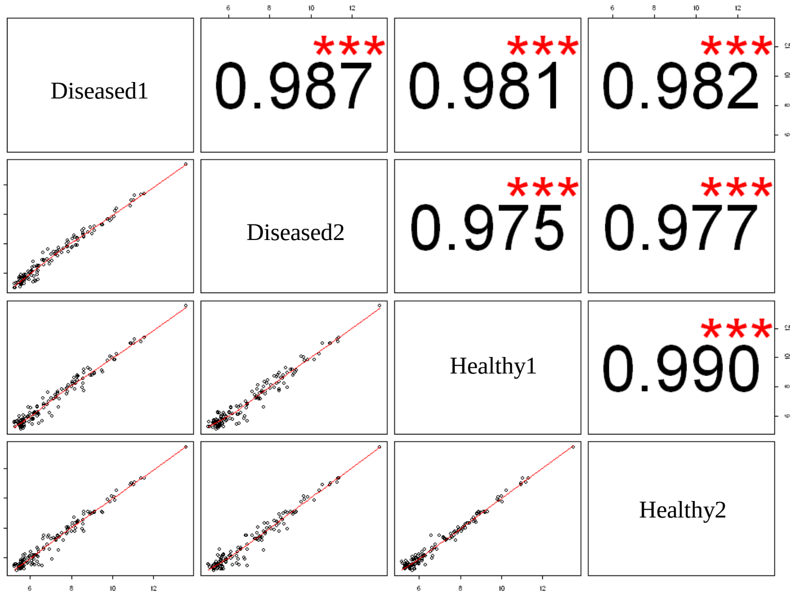

Figure 3: Scatter plots of all pairwise comparisons of probe intensities among four miRNA chips.

The upper and lower triangles of each square show the probe intensities along with regression estimation bisector lines and correlation coefficient among each pair. The maximum correlations are observed among Diseased:Diseased and Healthy:Healthy as expected. The highest correlation was determined among Healthy1 and Healthy2 chips.

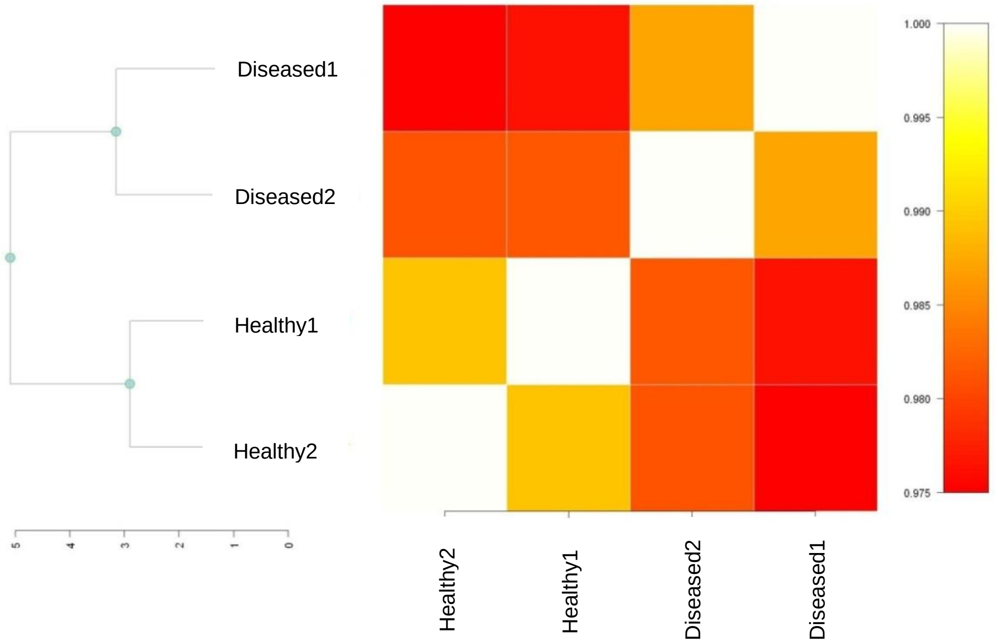

Figure 4: Hierarchical clustering heatmap among 4 miRNA microarrays from four different animals.

The arrays are well separated into two different groups i.e. Diseased and Healthy. The two major branches of the dendrogram represent disease and healthy animals with a correlation of 0.987 and 0.990 respectively. The high correlation values indicate that the samples were free from systematic biases.
